# Cortical dynamics and subcortical signatures of motor-language coupling in Parkinson’s disease

**DOI:** 10.1038/srep11899

**Published:** 2015-07-08

**Authors:** Margherita Melloni, Lucas Sedeño, Eugenia Hesse, Indira García-Cordero, Ezequiel Mikulan, Angelo Plastino, Aida Marcotti, José David López, Catalina Bustamante, Francisco Lopera, David Pineda, Adolfo M. García, Facundo Manes, Natalia Trujillo, Agustín Ibáñez

**Affiliations:** 1 Laboratory of Experimental Psychology and Neuroscience (LPEN), Institute of Cognitive Neurology (INECO), Favaloro University, Buenos Aires, 1854, Argentina; 2 Mental Health Group. School of Public Health. Universidad de Antioquia (UDEA), Medellín, 1226, Colombia; 3 Group of Neuropsychology and Conduct (GRUNECO), Faculty of Medicine, University of Antioquia (UDEA), Medellín,1226, Colombia; 4 Neuroscience Group, Faculty of Medicine, University of Antioquia (UDEA), Medellín, 1226, Colombia; 5 National Scientific and Technical Research Council (CONICET), Buenos Aires, 1033 Argentina; 6 UDP-INECO Foundation Core on Neuroscience (UIFCoN), Faculty of Psychology, Diego Portales University, Santiago, 8370076, Chile; 7 Faculty of Elementary and Special Education (FEEyE), National University of Cuyo (UNCuyo), Mendoza, 5502, Argentina; 8 SISTEMIC, Engineering Faculty, Universidad de Antioquia (UDEA), Medellín, 1226, Colombia; 9 Department of Research, Instituto de Alta Tecnología Médica de Antioquia, Medellín, 1234, Colombia; 10 Universidad Autónoma del Caribe, Barranquilla, 1234, Colombia; 11 Centre of Excellence in Cognition and its Disorders, Australian Research Council (ACR), New South Wales, 2109, Australia; 12 National University La Plata, Physics Institute, (IFLP-CCT-CONICET) La Plata, 1900, Argentina; 13 Physics Department, Universitat de les Illes Balears, Palma de Mallorca, 07122, Spain

## Abstract

Impairments of action language have been documented in early stage Parkinson’s disease (EPD). The action-sentence compatibility effect (ACE) paradigm has revealed that EPD involves deficits to integrate action-verb processing and ongoing motor actions. Recent studies suggest that an abolished ACE in EPD reflects a cortico-subcortical disruption, and recent neurocognitive models highlight the role of the basal ganglia (BG) in motor-language coupling. Building on such breakthroughs, we report the first exploration of convergent cortical and subcortical signatures of ACE in EPD patients and matched controls. Specifically, we combined cortical recordings of the motor potential, functional connectivity measures, and structural analysis of the BG through voxel-based morphometry. Relative to controls, EPD patients exhibited an impaired ACE, a reduced motor potential, and aberrant frontotemporal connectivity. Furthermore, motor potential abnormalities during the ACE task were predicted by overall BG volume and atrophy. These results corroborate that motor-language coupling is mainly subserved by a cortico-subcortical network including the BG as a key hub. They also evince that action-verb processing may constitute a neurocognitive marker of EPD. Our findings suggest that research on the relationship between language and motor domains is crucial to develop models of motor cognition as well as diagnostic and intervention strategies.

A growing body of research on motor cognition has revealed action language deficits in neurodegenerative disease^1^,^2^,^3^,^4^. Such impairments seem pervasive in Parkinson’s disease (PD)^5^,^6^,^7^, which is primarily caused by degeneration of dopaminergic neurons in basal ganglia (BG) structures^8^. Important evidence for this line of research has been obtained through the action-sentence compatibility effect (ACE) paradigm^9^, which assesses the integration of action-language comprehension and ongoing motor processes across compatible and incompatible trials^9^,^10^,^11^,^12^. The ACE is defined as shorter reaction times in compatible conditions (namely, when the action denoted by a verb involves a hand position similar to the one used to press the response button). Specifically, the ACE is abolished in early stages of PD (EPD)^3^,^13^ and in other cortico-subcortical motor diseases^14^. In this sense, performance on the ACE task has been proposed as a promising neurocognitive biomarker of PD^15^,^16^.

Motor-language coupling deficits in PD seem to result from damage to a BG-cortical motor network involving loops from frontotemporal areas to BG/thalamic structures and back to the cortex^3^,^14^,^16^. According to this model, BG impairment would disturb processing in this cortico-subcortical motor network, leading to action-verb deficits in EPD patients. Thus, this population may offer crucial information on the role of BG-cortical circuits in language processing.

Motor- and action-language-related disruptions in EPD involve abnormalities at a cortical level. EPD patients manifest aberrant cortical oscillation before and after the occurrence of a motor potential (MP), which likely represents activity of pyramidal neurons in the primary cortex and other motor structures, including the BG^17^,^18^. The MP is observed during motor execution^19^,^20^ and is time-locked to response onset (usually ranging from −100 to 50 ms around response onset). Specifically, EPD exhibit lower-amplitude beta-alpha event-related desynchronization response in contralateral sensorimotor cortices and other motor areas, which may serve as a biomarker of motor impairments^21^. Also, electrophysiological^10^,^22^ and intracranial^13^ recordings during the ACE task have shown a larger MP in the compatible condition, together with faster and more accurate responses.

At a subcortical level, the movement related cortical potential is partly generated by the BG up to even one second before the onset of movement^17^,^18^. The BG networks include pathways from and toward the motor and premotor cortices through cortico-striatal and thalamo-cortical loops^3^,^23^,^24^. BG damage is observed in PD^25^ and has been associated with verbal processing impairments.

Taken together, the above evidence suggests that action-language deficits in EPD involve multilevel disruptions in cortico-subcortical motor networks. To explore this possibility, we administered the ACE task to a group of EPD patients and examined potential cortical and subcortical markers of impaired performance. Specifically, we measured MP modulations and oscillatory connectivity patterns in connection with BG volume as revealed by voxel-based morphometry. Our results showed that EPD patients exhibited (a) behavioral impairments in the ACE task, accompanied by (b) a reduced cortical MP and (c) aberrant frontotemporal network activity. Also, (d) overall BG volume as well as specific BG atrophy regions predicted MP abnormalities during performance of the ACE task. Through this multi-dimensional approach, we offer unprecedented evidence of electrophysiological and structural signatures of ACE performance in EPD.

## Materials and Methods

### Participants

The sample comprised 14 EPD patients, diagnosed in accordance with the United Kingdom Parkinson’s Disease Society Brain Bank criteria^26^, and 13 healthy controls. Patients were excluded from the study if they exhibited non-parkinsonian neurological signs/symptoms or radiological structural brain abnormalities compatible with diagnoses other than PD. All remaining patients in the EPD group were under pharmacological treatment with levodopa or a dopamine agonist. Motor impairments and disease stages in EPD were measured with the Unified Parkinson’s Disease Rating Scale (UPDRS)^27^ and the Hoehn and Yahr (H&Y) scale^28^, respectively. Control participants were recruited via a database of healthy volunteers from an ongoing project. None of these subjects had a history of neurodegenerative disease, psychiatric disorders, or drug abuse.

Both groups were matched for age, handedness, level of education, and proportion of male to female participants. They also completed an evaluation including dementia measures and a verbal processing task. The groups’ characteristics, clinical and demographic data are summarized in Table 1 and thoroughly discussed in the Supplementary Data (*Participants* subsection of Material and Methods section). All participants read and signed an informed consent form prior the beginning of the study and all experiments were performed in accordance with relevant guidelines and regulations of the Declaration of Helsinki before beginning the study. All experimental protocols of this study were approved by the ethics committee of the Institute of Cognitive Neurology.

### Kissing and Dancing Test

Access to action-verb semantics was assessed trough the Kissing and Dancing Test (KDT)^29^. The test consists of 52 triads of images, each composed of a cue picture at the top and two options at the bottom. Participants must point to the option that is most closely related to the cue picture. As shown in previous studies^13^,^15^, the KDT is sensitive to subtle action-semantic deficits in EPD (see Supplementary Data, *Kissing and Dancing Test* subsection of Material and Methods). Results for the KDT are presented in Table 1.

### ACE task

We used the ACE paradigm following previously reported procedures^13^,^15^. Participants listened to sentences involving actions typically performed with an open hand (OH [*n* = 52]) or a closed hand (CH [*n* = 52]), as well as neutral sentences (N [*n* = 52]) denoting non-manual actions. In the first two sets, the verb denoting the manual action appeared in sentence-final position. Upon comprehension of each sentence, participants pressed a button with their dominant hand in a pre-assigned shape (open or closed). The combination of response type and sentence type generates compatible (OH sentence and OH response, or CH sentence and CH response), incompatible (OH sentence and CH response, or vice versa), and neutral (neutral sentence with either response) trials. Hand-shapes were counterbalanced across blocks. Each sentence list was divided into two sub-lists of 26 sentences. One sub-list from each pair was randomly assigned for each participant to a response block (OH and CH responses). After the task, all participants completed an offline questionnaire assessing comprehension of the sentences. As in previous reports^13^,^15^, the global ACE score (Table 1) was defined as the difference between the mean reaction time of the incompatible and the compatible conditions. For further details, see Supplementary Data (*ACE Task* subsection of Material and Methods section).

### Electroencephalography recordings

Electroencephalography (EEG) signals were recorded online with a Synamps Neuroscan 64-channel system at 1000 Hz sampling rate. Analogue filters were 0.03 and 100 Hz. For analysis of event-related potentials (ERPs), a digital band pass filter between 0.5 and 30 Hz was applied offline to remove unwanted frequency components. The reference was set to link mastoids. Two bipolar derivations were employed to monitor vertical and horizontal ocular movement electro-oculogram. Epochs were selected from continuous data, from −500 ms to 1000 ms for hand-response-locked segments. Eye movements or blink artifacts were corrected with independent component analysis, and remaining artifacts were rejected offline from trials that contained voltage fluctuations exceeding ±200 μV, transients exceeding ±100 μV, or electro-oculogram activity exceeding ±70 μV.

### MRI recordings

All participants were scanned in a 1.5 T Phillips Intera scanner equipped with a standard head coil. A T1-weighted spin echo sequence was used to generate 120 contiguous axial slices (TR = 2300 msec; TE = 13 msec; flip angle = 68°; FOV = rectangular 256 mm; matrix size = 256 × 240 × 120; and slice thickness = 1 mm).

### Data analysis

Numerical demographic data were compared via independent-sample *t* tests, while categorical variables (e.g., gender) where analyzed through chi-square tests. Comparison of neuropsychological data between the groups was performed using ANOVA. Effects sizes were calculated through partial eta (η_p_^2^).

### Behavioral measures

Behavioral performance in the ACE task was analyzed through repeated measures ANOVA, with group as the between-subject factor (EPD and controls) and category as the within-subject factor (compatible, incompatible, and neutral). Reaction times were calculated for each subject in each condition (compatible, incompatible, and neutral). Responses with reaction times beyond +2.5 standard deviations were considered outliers and excluded from the analysis. A global ACE score^13^,^15^ was obtained by subtracting the mean of the compatible trials from that of the incompatible trials. Post hoc contrasts were calculated with Tukey’s HSD test. Statistical significance was defined as p < 0.05.

### ERPs

**Figure 1:**
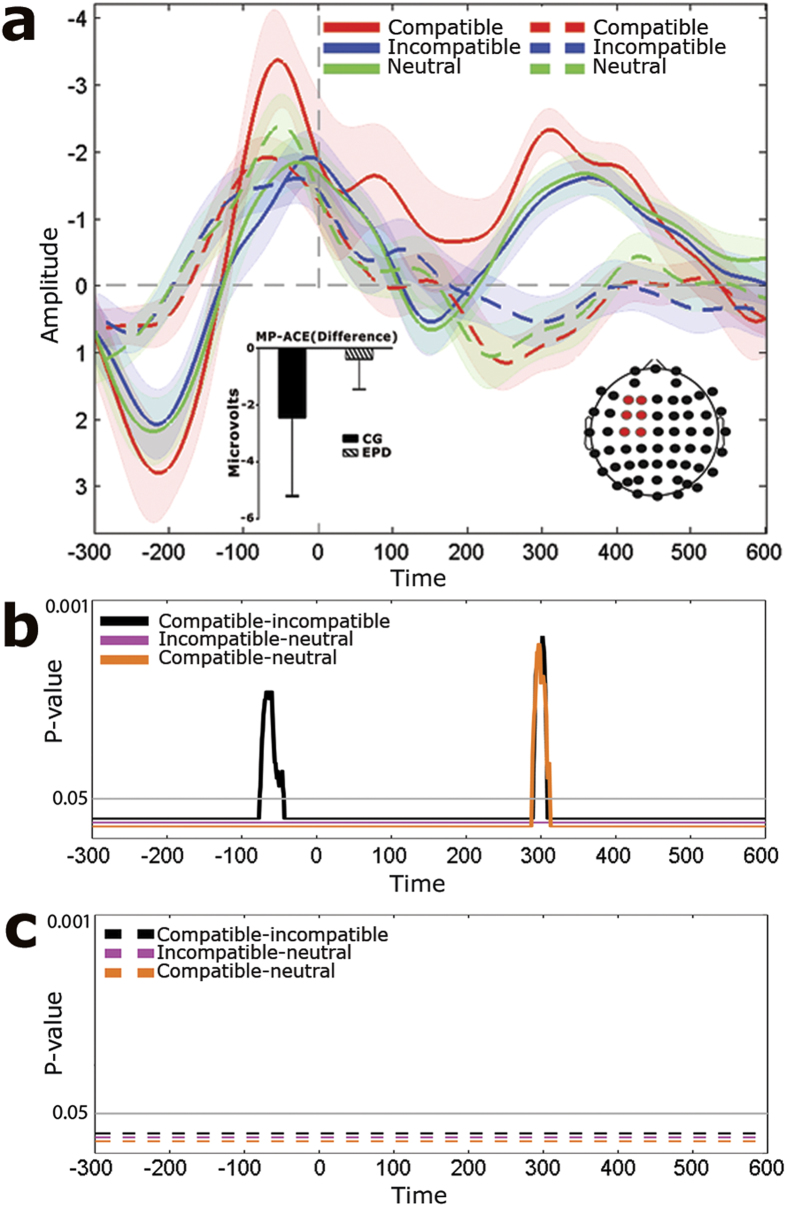
MP modulations. (**A**) ERPs of compatible, incompatible, and neutral categories for both controls (continuous line) and EPD (dotted line). Shadowed bars around potentials indicate s.e.m. Channel locations highlighted in red shows the regions of interest where ERPs were obtained. The bar graph shows the mean MP-ACE global score (compatible-minus-incompatible trials) for EPD and controls. (**B**) Significant differences between conditions in controls based on the Monte Carlo permutation test, throughout the ERP time window. (**C**) Non-significant differences between conditions in EPD, throughout the ERP time window. In B & C, waveforms depicts the *p* values resulting from permutations and after bootstrapping for each comparison. The horizontal line indicates when a *p* value crosses the threshold of significance.

EEG offline processing and analysis was conducted on Matlab software. Artifact-free epochs were averaged to obtain the ERPs. ERP waveforms were averaged separately for each experimental condition. The MP was observed at canonical sites (around C3). Based on a previous report^10^, ERP analysis focused on a region of interest including six electrodes around the maxima of MP (electrodes 8, 9, 17, 18, 26, 27; see Fig. 1A). This analysis is consistent with previous reports for maxima location of motor responses^19^. Differences among categories and groups were assessed for significance via Monte Carlo permutation tests with bootstrapping^30^ (see Supplementary Data, *ERPs analysis* subsection of Material and Methods). To evaluate the association between MP and structural neuroimaging data (see below), a composite MP-ACE score was built by subtracting the ERP waveforms from incompatible-minus-compatible categories and mean averaged in the time windows yielding significant differences (see below).

### Functional connectivity

**Figure 2:**
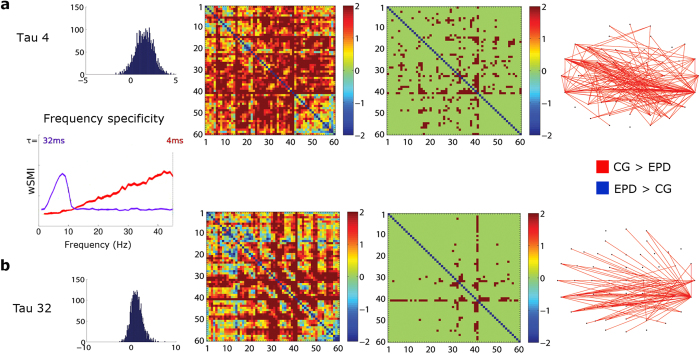
Functional connectivity during the ACE task. Global broadcasting of information across distant cortical regions (wSMI). Two tail *t*-test on the wSMI matrices of each group were obtained by subtracting the correlation matrices of the incongruent and congruent conditions. The Frequency Specificity Graph shows the sensitivity of wSMI to pure-frequency signals. The value of τ makes the wSMI measure sensitive to different frequency ranges (τ = 4 ms is specific for frequencies among 11–40 Hz and τ = 32 ms is specific for frequencies ranging between 1 and 11 Hz). **(A)** Analysis for tau 4 ms (>11 Hz): (i) histogram showing the number of occurrences (y axis) of the *t* values (x axis); the distribution of these values exhibits a positive trend, indicating that information sharing is larger for controls than EPD patients; (ii) correlation matrix of raw *T* value; (iii) masked correlation matrix: *T* values were corrected with an alpha level set at *p* < 0.01; non-significant values were assigned a 0; (iv) connectivity map of significant connections only across the scalp indicating that controls presented higher information sharing at frontotemporal regions. **(B)** Analysis for tau 32 ms (specific for 1–11 Hz): (i) histogram showing the number of occurrences (y axis) of the *t* values (x axis); the distribution of these values exhibits a positive trend, indicating that information sharing is larger for controls than EPD patients; (ii) correlation matrix of raw *T* value; (iii) masked correlation matrix: *T* values were corrected with an alpha level set at *p* < 0.001; non-significant values were assigned a 0; (iv) connectivity map of significant connections only across the scalp indicating that controls presented higher information sharing mainly at bilateral temporal regions.

The ACE is hypothesized to require integration of information among frontotemporal regions^3^,^13^. We explored this conjecture using a novel measure of integration and global broadcasting of information across distant cortical regions, called Weighted Symbolic Mutual Information (wSMI) (for details, see Supplementary Data, *Connectivity* subsection of Material and Methods, and^31^). The wSMI measure assesses the extent to which two signals present nonrandom joint fluctuations (sharing information), characterized by (a) fast and robust estimation of the signals’ entropies, (b) detection of nonlinear coupling, and (c) absence of spurious correlations between EEG signals arising from common sources^31^. EEG signals were first transformed into a series of discrete symbols defined by the ordering of k time samples segregated by a temporal separation τ. Analysis was restricted to a fixed symbol size (k = 3) and two different values of τ (τ = 4 and 32 ms between time samples). We focused our analysis on τ = 4 and 32 ms because they are specific for two different frequency ranges: slow oscillations related to MP (assessed with τ = 32 ms, 1–11 Hz) and a broad band of higher frequencies (τ = 4 ms, 11–40 Hz). Previous studies on PD have reported affectations in the former^21^ and aberrant oscillations in the latter^32^,^33^ (see Supplementary Data, *Connectivity* subsection of Material and Methods)^31^. To calculate wSMI for each pair of transformed EEG signals, we estimated the joint probability of each pair of symbols. To reduce spurious correlations between signals, the joint probability matrix was multiplied by binary weights. The weights were set to zero for pairs of identical symbols, which could be elicited by a unique common source, and for opposed symbols, which could reflect the two sides of a single electric dipole. We used the increment of sharing information between ACE conditions by subtracting the correlation matrix of the incongruent condition from that of the congruent condition. This measure was used to investigate connectivity differences between both groups (EPD and controls, Fig. 2). Also, two-tail *t* tests were performed between correlation matrices of each group. Alpha levels were set at *p* < 0.01.

### Structural neuroimaging

**Figure 3:**
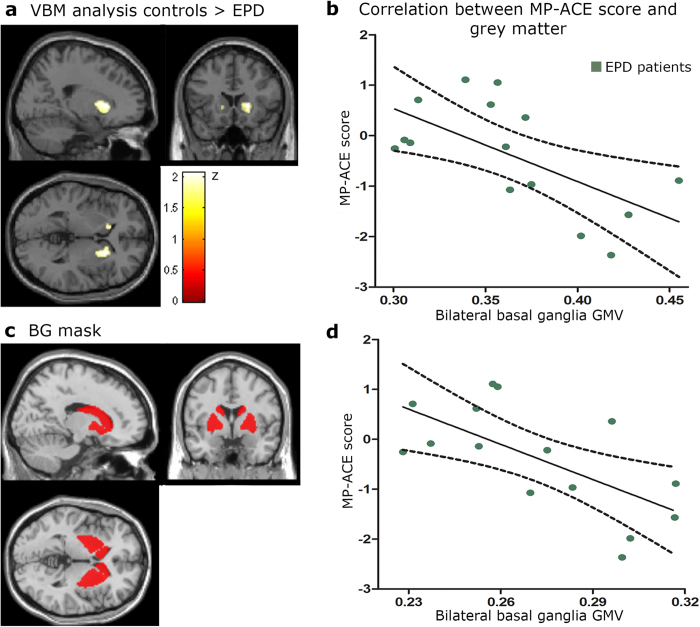
Voxel-based morphometry results and correlation between BG GMV and ACE performance in EPD. (**A**) Cluster of significant GMV atrophy of the bilateral BG (caudate, putamen, globus pallidus) of EPD patients compared to controls. (**B**) ERP-structural volume correlation between the mean GMV of the significant atrophy cluster and MP-ACE global score in EPD (*r* = −.63, *p* = .012). (**C**) BG mask obtained from the AAL atlas. (**D**) ERP-structural volume correlation between the mean of GMV of bilateral BG and MP-ACE score in EPD (*r* = −.65, *p* = .009).

To investigate the association between MP and overall BG volume, we assessed the total GMV of bilateral BG. Also, to examine association between MP and BG atrophy, we extracted the cluster of significant atrophy in the bilateral BG using voxel-based morphometry. We were thus able to explore whether the cortical ACE modulation (MP-ACE score) was associated with (a) overall BG volume and (b) specific atrophy areas within the BG. Images were preprocessed on the DARTEL Toolbox according to previously described procedures^34^ and modulated 8-mm full-width half-maximum kernel-smoothed^35^. Images were normalized to the MNI space and analyzed with general linear models for second-level analyses on SPM-8 software. To compare grey matter volumes (GMV), we used a two-sample comparison between EPD and controls (*p* < 0.05, extent threshold = 50). We then extracted the GMV from the significant atrophy cluster of the bilateral BG (caudate, putamen, and globus pallidus; see Fig. 3a) using MarsBaR region of interest toolbox^36^. Correlation coefficients were calculated between the mean GMV of the significant atrophy cluster and the MP-ACE score (Fig. 3b). In addition, we calculated the mean GMV of bilateral BG using a predefined mask (which includes caudate, putamen, and globus pallidus) from the AAL atlas^37^ (Fig. 3c). Finally, we correlated the mean GMV of bilateral BG and the MP-ACE score (Fig. 3d).

## Results

Table 1 summarizes demographic, clinical, and action-language data for EPD patients and controls. No differences among groups were observed in age, education, gender or handedness (all participants were right-handed). As expected, the groups differed in action-language performance. Relative to controls, the EPD group showed deficits in both the KDT [*F* (1, 23) = 5.43; *p* = .028, η_p_^2^ = .0191] and the ACE task (global score: [*F* (1, 25) = 9.57, *p* = .004, η_p_^2^ = .276 )] (for further statistical analysis, see Supplementary Data, *Demographic and language evaluation* subsection of Results section). These results replicate previous findings^13^,^15^.

### Motor potential modulations during the ACE task

In the control group, the MP (Fig. 1a) showed a significant ACE (Fig. 1b), with more negative amplitudes in compatible than incompatible trials. In the MP time window, the modulations elicited by neutral trials did not significantly differ from those of either compatible or incompatible trials. The compatible condition also yielded greater negativity that both the incompatible and neutral conditions in a scalp and time window consistent with the re-afferent potential (RAP, 220 to 480 ms)^10^.

Instead, EPD patients (Fig. 1a) exhibited no significant differences between compatible and incompatible trials (Fig. 1c), revealing absent ACE-induced modulations. Also, relative to controls, EPD patients showed reduced amplitudes for each condition in time windows established before the MP and after the RAP (Figure S1 in Supplementary Data).

Moreover, parametric and non-parametric correlations between MP-ACE score and UPDRS motor subscale in patients were significant upon removal of two outlier values (Pearson’s *r* = .62, *p* = .030; Spearman’s *r* = .60, *p* = .036; Figure S2 in supplementary data).

### Connectivity

Analysis of global broadcasting of information across distant cortical regions trough wSMI revealed higher frontotemporal information sharing in controls at higher frequencies (11–40 Hz, τ = 4, Fig. 2a). All p values under 0.01 were considered significant. Similar results were obtained for τ = 32 (1–11 Hz, Fig. 2b), only in more bilateral temporal regions. In this case, all p values under 0.001 were considered significant.

### Voxel-based morphometry

#### BG atrophy in EPD patients

Compared to controls, EPD exhibited significant atrophy in bilateral BG structures, including the putamen, the caudate nucleus, and the globus pallidus (Table 2, Fig. 3a).

#### Associations between GMV in BG and MP-ACE

In both groups, cortical measures of ACE (MP-ACE) were associated with GMV in bilateral BG structures, including the putamen, the caudate nucleus, and the globus pallidus (*r* = −.51, *p* = .008). Moreover, in EPD patients, greater atrophy correlated with worse MP-ACE global scores (*r*_*s*_ = −.63, *p* = .012; Fig. 3b).

An association of bilateral BG volume and MP-ACE was evidenced when considering both groups through region-of-interest analysis (*r* = −.45, *p* = .023). Furthermore, as shown in Fig. 3d, patients’ mean GMV in bilateral BG was associated with ACE; the lower the GMV, the lower the MP-ACE score (*r* = −.65, *p* = .009).

## Discussion

PD has long been misconceived as a purely motor disease^1^. However, multiple reports in the last two decades have indicated that impairments of movement and cognition are not separate phenomena^38^,^39^. Instead, motor disorders typically involve deficits in high-order domains, such as action language^6^,^40^,^41^,^42^,^43^. In particular, recent studies employing the ACE paradigm in PD patients^13^,^15^ suggest that motor-language coupling depends on a distributed network involving BG structures. Selective action-language impairments in EPD would result from damage to such a network. To our knowledge, this is the first report providing multilevel evidence of cortico-subcortical markers of impaired ACE performance. Specifically, disturbances in a BG-frontotemporal network seem to underlie such deficits in EPD. The present data support a model of motor cognition which highlights the contributions of complex BG circuitry to action-verb processing. These findings pave the way to redefine neuropsychological assessment in EPD in the pursuit of more successful intervention strategies.

Regarding cortical dynamics of ACE, our behavioral results replicate findings^13^,^15^, as the ACE has previously been shown to be impaired in EPD patients. Importantly, such deficits were accompanied by distinct ERP patterns. In controls, as previously reported^10^, the MP and the RAP presented larger amplitudes in the compatible than in the incompatible condition (Fig. 1a). Conversely, EPD patients exhibited no such cortical modulations during the task (Fig. 1c). The general reduction of MP in EPD (Figure S1) replicates classical work of evoked motor activity during self-paced movements in this population^44^. These results constitute the first demonstration of joint behavioral and electrophysiological disturbances in motor-language coupling in EPD.

Global information sharing across distant cortical areas during the ACE task was examined through wSMI^31^. This measure provides information on the brain’s capacity to integrate information, as observed in high-level cognitive processes such as language processing^45^. In controls, as compared with EPD patients, incompatible ACE trials yielded higher information sharing in frontotemporal regions at higher frequencies (τ = 4, 11–40 Hz) (Fig. 2a). The same was true at lower frequencies (τ = 32, 1–11 Hz), but more restricted to bilateral temporal regions (Fig. 2b). The present results suggest that functional connectivity can be modulated by cognitive load during motor-language coupling, consistent with previous research showing activation increases as a function of complexity^46^. In similar contextual congruency tasks, incongruent conditions elicited greater activation levels in core language areas (left posterior middle temporal and inferior frontal regions)^47^,^48^. Such a pattern of increased frontotemporal connectivity was abolished in EPD.

Interestingly, functional connectivity between cortical areas and the BG is reduced during self-initiated movements in PD^49^,^50^,^51^, suggesting a possible marker of motor/cognitive dysfunction^51^. Moreover, in PD, aberrant oscillatory activity (diminished beta/alpha desynchronization before and after the movement) plays an important role during motor control deficits and concomitant movement difficulties^21^. Compatibly, studies of high-frequency oscillations have revealed dopamine and movement-modulated gamma activity in the subthalamic nucleus^52^ and internal globus pallidus^53^. Moreover, a cross-frequency relation between beta and high-frequency oscillations has been observed as a correlate of motor symptoms in PD^33^,^52^. We suggest that reduced frontotemporal information exchange at lower and high frequencies in EPD would be triggered by damage to BG-striatal circuits required for an adequate motor response. Our behavioral and electrophysiological (MP and connectivity) measures provide convergent evidence of the impaired brain dynamics underlying the abolished ACE in EPD.

Action-language deficits in PD have been proposed to reflect compromise of a cortico-subcortical network critically involving BG structures^13^. In this study, BG volume was associated with the cortical markers of ACE in both EPD patients and matched controls. First, we observed atrophy of bilateral BG structures (putamen, caudate nucleus, globus pallidus) in EPD compared to controls (Fig. 3a). Also, MP-ACE activity was associated with BG volume (atrophy regions and overall volume). Specifically, worse performance on the ACE task was correlated with smaller GMV in the BG (Fig. 3). Importantly, the association between MP-ACE global score and BG GMV was stronger in EPD patients, emphasizing the critical role of the BG in ACE disruptions.

Moreover, modulation of the MP during ACE performance was associated with motor symptoms as assessed through the UPDRS: the lower the cortical modulation, the higher the motor symptoms. This pattern suggests a connection between the cortical dynamics of action language and motor symptomatology.

Overall, these results constitute the first demonstration of convergent temporal-dynamics and structural abnormalities underlying impaired motor-language coupling in EPD. These findings confirm previous results suggesting that in EPD, BG atrophy compromises behavioral performance and cortical dynamics during motor-language integration.

A number of limitations must be recognized in this study. The modest sample size may have affected statistical results. Yet, the effects obtained were robust, and other studies using electroencephalography and voxel-based morphometry^54^,^55^ have also yielded robust findings with similar or even smaller sample sizes. In addition, the association between motor symptomatology and MP modulation during ACE should be taken with caution since (a) our sample size was small, (b) the UPDRS scale was not administered to the control group, and (c) correlations are reported without two outliers. Also, note that all assessments were conducted during the ‘on’ state of medication.

Given the reduced sample size and unavailable data regarding medication, we were not able to assess the effect of medication in our task. Yet, since levodopa seems to improve verbal processing in a percentage of PD subjects^56^, ACE impairments could hardly be attributed medication effects. In this sense, future studies should compare EPD patients both on and off medication.

Language studies in subclinical PD patients with genetic vulnerability could also shed light on early cognitive markers of this disease. Another limitation is that the low and high frequency ranges covered by the two τ values chosen for this study were too widespread. In fact, these bands are too gross to detect standard functional brain oscillations –and specific frequencies are associated with different physiological meanings. Nevertheless, abnormal oscillatory activity in PD has been reported in a wide range of relatively low (delta, theta, and alpha) and high (beta and gamma) bands. Thus, we decided to use a measure of information integration that includes broad frequency bands covering the lower (τ = 32) and the higher (τ = 4) frequency bands previously reported as affected in PD. This is also relevant, given that cross frequency coupling between low and high frequency oscillations are observed in PD^33^, suggesting that an extended broadband approach may be helpful to characterize these aberrant oscillations.

Our findings have clinical implications. Action-language assessment could be incorporated in a new agenda for testing patients with fronto-striatal damage. Such a domain could complement the classical focus on memory and executive functions. In this sense, the ACE task could aid effective diagnosis through early detection of linguistic alterations and related neurocognitive markers even before the occurrence of other impairments, thus paving the way for timely application of cognitive stimulation programs^16^. Moreover, this prospective assessment model could be extended to other neurodegenerative motor diseases.

The BG are currently acknowledged as a key substrate of high-order cognitive domains, such as executive functions and language. The variety of domains associated with the BG reflects their complex organization and multiple circuitries, including pathways from and toward the motor and premotor cortices through cortico-striatal and thalamo-cortical loops^23^,^24^. A network involving the BG, the thalamus, and Broca’s area has been implicated in language processing^57^. Similarly, Cardona *et al.*^3^ proposed a model in which the anatomic substrate for action-language processing would consist in a BG-frontotemporal loop, whose disruption would impair ACE performance. Building upon such findings^3^,^13^, we set forth a model distinguishing two networks underlying action-language processing: a motor circuit and a semantic circuit, both crucially related to BG structures engaged by the ACE task. This model aligns with embodied cognition approaches^58^, which propose that semantic and conceptual information is grounded in sensorimotor experience. In EPD, ACE impairments may reflect a disruption in the integration of motor and linguistic information caused by BG damage and concomitant compromise of frontotemporal networks. The present results support the model by Cardona *et al.* Moreover, they allow us to *extend* it by adding a temporal-dynamic dimension that complements the original anatomic proposal. Indeed, proper functioning of the BG-frontotemporal loop subserving action language seems to involve adequate electrophysiological and oscillatory activity. Our findings warrant the postulation of a multidimensional model of motor-language coupling, which brings together behavioral (ACE), ERP (MP), functional connectivity (wSMI), and anatomical (VBM) data. At its most basic, the model would imply the following dynamics: adequate motor-language coupling through the BG-frontotemporal loop involves modulations of early, automatic (MP) and later (RAP) electrophysiological activity, as well as high information-sharing levels among remote cortical regions, especially during cognitively demanding processes. The above mechanisms crucially depend on the integrity of gray matter in BG structures. Further conceptual grounding during motor-language integration would be afforded by additional striatal-temporal-thalamic loops.

More generally, this study shows the benefits of directly exploring the interplay of varied dimensions in a single neurocognitive study. The formulation of theories which tie together spatial and temporal data to predict emergent properties of the brain has been recently acknowledged as a key challenge for neuroscience^59^. Several models have long sought to integrate data from varied levels by considering data from multiple studies. While this approach is certainly useful, subject-, task-, and stimulus-related differences across the studies may undermine the success of their integration. This caveat can be partly circumvented by conducting multidimensional experiments, in which behavioral performance, electrophysiological correlates, network segregation patterns, and gray matter density are jointly assessed in the same sample performing the same task.

To conclude, our findings highlight the importance of BG structures and their influence on frontotemporal networks for motor-language coupling. The clinical and theoretical implications outlined above should be more deeply explored in EPD and extrapolated to other neurodegenerative diseases involving comparable action-language compromise following BG damage (e.g., progressive supranuclear palsy, Huntington’s disease, cortico-basal degeneration).

## Additional Information

**How to cite this article**: Melloni, M. *et al.* Cortical dynamics and subcortical signatures of motor-language coupling in Parkinson's disease. *Sci. Rep.* **5**, 11899; doi: 10.1038/srep11899 (2015).

## Supplementary Material

Supplementary Data

## Figures and Tables

**Table 1:** Clinical, demographic, and behavioral (action language) results.

		EPD	CG	EPD vs CG
		n = 14	n = 13	*p*value
Demographic Variables	Age (years)	56.07 (11.20)	54.72 (10.03)	.737
	Education (years)	11.85 (4.72)	11.54 (0.52)	.421
	Gender (F : M)	7:7	6:5	.841
Clinical Variables	Years diagnosed	22.00 (12.42)	N/A	N/A
	UPDRS III	2.39 (0.65)	N/A	N/A
	H&Y	6.53 (3.46)	N/A	N/A
KDT		91.07 (2.05)	96.67 (0.69)	.002
ACE-RT		27.05 (50.63)	−207.93 (56.60)	.004

EPD and CG values are provided in means (SD). Abbreviations: CG, Control group; EPD, early Parkinson’s disease; UPDRS, Unified Parkinson’s Disease Rating Scale; H&Y, Hoehn and Yahr’s scale; NA, not applicable; KDT, Kissing and Dancing Test; ACE-RT, global reaction-time scores on the ACE task.

**Table 2:** Regions of significant atrophy (local maxima) in EPD compared with CG.

Region	X	Y	Z	Cluster k	Peak t	Peak z
Putamen right	21	18	1,5	593	2,07	1,97
Caudate nucleus	−13,5	21	−5,68	117	1,92	1,85
Globus Pallidus	24	−7,5	−3	8	1,82	1,76

All p < 0.05.
