# Supplementary material for: Cortical dynamics and subcortical signatures of motor-language coupling in Parkinson’s disease
Source: Sci Rep. 2015 Jul 8;5:11899. doi: 10.1038/srep11899 (PMC4495549; doi:10.1038/srep11899)
Supplement: Supplementary Data [file srep11899-s1.pdf]

Supplementary Data

Cortical dynamics and subcortical signatures of motor-language coupling in Parkinson's disease

Margherita Melloni[a,e,f], Lucas Sedeño[a,e,f], Eugenia Hesse[a,e,f], Indira García-Cordero[a,e,f], Ezequiel Mikulan[a,e,f], Angelo Plastino[e,l,m], Aida Marcotti[a], José David López[h], Catalina Bustamante[i], Francisco Lopera[d], David Pineda[c,d], Adolfo M. García[a,e,f,g], Facundo Manes[a,e,f,k], Natalia Trujillo [b,c,d], Agustín Ibáñez[a,e,f,j,k]*

[a] Laboratory of Experimental Psychology and Neuroscience (LPEN), Institute of Cognitive Neurology (INECO), Favaloro University, Buenos Aires, 1854, Argentina.

[b] Mental Health Group. School of Public Health. Universidad de Antioquia (UDEA), Medellín, 1234 Colombia.

[c] Group of Neuropsychology and Conduct (GRUNECO), Faculty of Medicine, University of Antioquia (UDEA), Medellín,1234, Colombia.

[d] Neuroscience Group, Faculty of Medicine, University of Antioquia (UDEA), Medellín, 1234, Colombia.

[e] National Scientific and Technical Research Council (CONICET), Buenos Aires, 1033 Argentina.

[f] UDP-INECO Foundation Core on Neuroscience (UIFCoN), Diego Portales University, Santiago, 8370076, Chile.

[g] Faculty of Elementary and Special Education (FEEyE), National University of Cuyo (UNCuyo), Mendoza, 5502, Argentina.

[h] SISTEMIC, Engineering Faculty, Universidad de Antioquia (UDEA), Medellín, 1234, Colombia

[i] Department of Research, Instituto de Alta Tecnología Médica de Antioquia, Medellín, 1234, Colombia.

[j] Universidad Autónoma del Caribe, Barranquilla, 1234, Colombia.

[k] Centre of Excellence in Cognition and its Disorders, Australian Research Council (ACR), New South Wales, 2109, Australia.

[l] National University La Plata, Physics Institute, (IFLP-CCT-CONICET) La Plata, 1900, Argentina.

[m] Physics Department, Universitat de les Illes Balears, Palma de Mallorca, 07122, Spain.

* Corresponding author: Agustín Ibáñez, Ph.D., Laboratory of Experimental Psychology and Neuroscience (LPEN), Institute of Cognitive Neurology (INECO) and CONICET. Pacheco de Melo 1860, Buenos Aires, Argentina. Phone/Fax: +54 (11) 4807-4748. aibanez@ineco.org.ar

Material and Methods

Participants

EPD patients who met the UK Parkinson's Disease Society Brain Bank criteria [1] were evaluated with the Unified Parkinson's Disease Rating Scale (UPDRS) part III [2] and stages I and II of the Hoehn and Yahr scale [3]. Mean age for the EPD group was 56.07 (±11.20) years. All selected patients had bilateral or unilateral disease onset in their dominant right hand. Assessment was conducted during the 'on' state of the medication. Since levodopa seems to improve verbal processing in a percentage of PD subjects [4], any observed impairment of ACE or verbal processing cannot be explained by medication. Additional requisites for inclusion within the EPD group were disease duration of fewer than five years and absence of motor complications from levodopa [5,6]. The control group was composed of healthy volunteers with functional independence and IQs above 90 –as determined by the vocabulary and similarities subtests of the Wechsler Abbreviated Scale of Intelligence (WASI) [7].

Kissing and Dancing Test

Bak and Hodges [8] created this test in order to assess semantic association of action verbs. It comprises 52 triads of images depicting motor actions. Each triplet is composed of a cue action-picture and two semantically related pictures. Participants are required to point to the picture that is most closely related to the cue picture.

ACE Task

Both hands of each participant were positioned in the required shape to control for possible bilateral hand interference, given that posture has been shown to modulate semantic processing [9-12]. Participants completed a five-trial training session to become familiar with the task. Each trial began with an ocular fixation cross appearing at the centre of the monitor 300 ms before the beginning of the sentence and disappearing 800 ms after the response. The interstimulus interval was set at 150 ms. Stimuli were simple Spanish sentences with a critical third-person verb in *pretérito indefinido del indicativo* (simple past tense), located in sentence-final position.

The sentence lists used in the compatible and incompatible conditions were controlled for relevant linguistic variables, including transitivity, situation aspect, clause content, final target-word frequency, predictability, prototypicality (how well the pertinent hand-shape represented the manual action encoded by the sentence), and degree of manual specificity (the manual aperture or closure for each sentence). Note that neutral sentences are more predictable in this paradigm, thus eliciting faster reaction times. See Aravena et al. [13] for details about predictability effects.

Mean sentence duration was 4.57 s (*SD* = .16 s). Audio files were edited so that each trial was preceded and followed by silence periods of 400 ms and 200 ms, respectively. Mean onset-time of the target verb within the sentences was 4.05 s (*SD* = .06; 2.92 s minimum, 5.64 s maximum). Trials were uniformly distributed over the three sentence conditions in a counterbalanced list to ensure that the same condition did not appear more than two times consecutively. See Aravena et al. [13] for more details on stimuli features and validation.

ERP analysis

Statistical analysis of ERPs during the ACE task was performed at MP windows using Monte Carlo permutation tests with bootstrapping [14]. The combined data from each condition (e.g., the compatible and incompatible trials) underwent a random partition, and a *t*-test was calculated. This process was repeated 1,000 times to construct the *t*-value distribution under the null hypothesis. The null hypothesis is rejected if an obtained *t*-value is greater than the most extreme 1% of the distribution (e.g., $p < .01$). The significant window obtained in the permutation analysis was selected to calculate the MP-ACE score (subtracting the waveforms from incompatible-minus-compatible categories and mean averaged in in this significant time window: -80 to 40 ms).

Connectivity

The weighted Symbolic Mutual Information (wSMI) measure presents three main advantages. First, it looks for qualitative or "symbolic" patterns of increase or decrease in the signal, which allows a fast and robust estimation of the signals' entropies. The symbolic transformation depends on the length of the symbols (here, k = 3) and their temporal separation (here, τ = 4, or 32 ms, [15]). Second, wSMI makes few hypotheses on the type of interactions and provides an efficient way to detect non-linear coupling. Third, wSMI weights discard the spurious correlations between EEG signals arising from common sources and favor non-trivial pairs of symbols [16]. EEG signals were first transformed into a series of discrete symbols defined by the ordering of k time samples separated by a temporal separation τ (Figure 2A of main manuscript). Analysis was restricted to a fixed symbol size (k = 3) and two different values of τ (τ = 4, 32 ms between time samples). Low-pass filters at corresponding frequencies (80, and 10 Hz for τ = 4 and 32 ms respectively) were used to avoid aliasing artifacts. The wSMI was estimated with a joint probability matrix multiplied by binary weights. These weights were set to zero for

pairs of (a) identical symbols and (b) opposed symbols that could be elicited by a unique common source or the two sides of a single dipole, respectively. The distance separating EEG channels was calculated along a straight line using default electrode coordinates.

Results

Demographic and language evaluation

Comparisons between EPD patients and controls revealed no significant differences in age [t (23) = 0.31, p = .758], formal education [t (23) = 0.14, p = .885] or gender [x^2 (1, N = 27) = .51, p = .821)].

KDT

The KDT is designed to detect impairments in action semantics. Significant group differences were observed [F (1, 23) = 5.43; p = .028, η_p^2 = .191]. The KDT score (% of correct responses) was significantly lower for EPD patients (M = 91.07, SD = 2.5) than controls (M = 96.67, SD = 0.69). This result is consistent with reports of action-verb processing deficits in PD [17-19].

ACE is impaired in EPD

A significant effect of group [F (1, 25) = 5.95, p = .022] was observed. EPD patients showed longer reaction times than controls in the three conditions. No significant effect of Compatibility was observed [F (2, 50) = 1.56, p = 0.218)]. However, we found a strong interaction of Group X Compatibility [F (2, 50) = 3.41, p = .040]. A *post hoc* analysis (MS = 3769; df = 28.37) showed an ACE in controls: incompatible trials elicited longer reaction times than compatible trials (p = .009). A significant difference was also observed between compatible and neutral trials (p = .030). No significant effect was found between

incompatible and neutral trials (*p* = .629). Conversely, we found no ACE in EPD patients. Here, reaction times were similar among the three conditions: no differences were observed between compatible and incompatible trials were observed (*p* = .694), neutral and compatible trials (*p* = .382), or neutral and incompatible trials (*p* = .628) (see means and *SD*s in Table 1S).

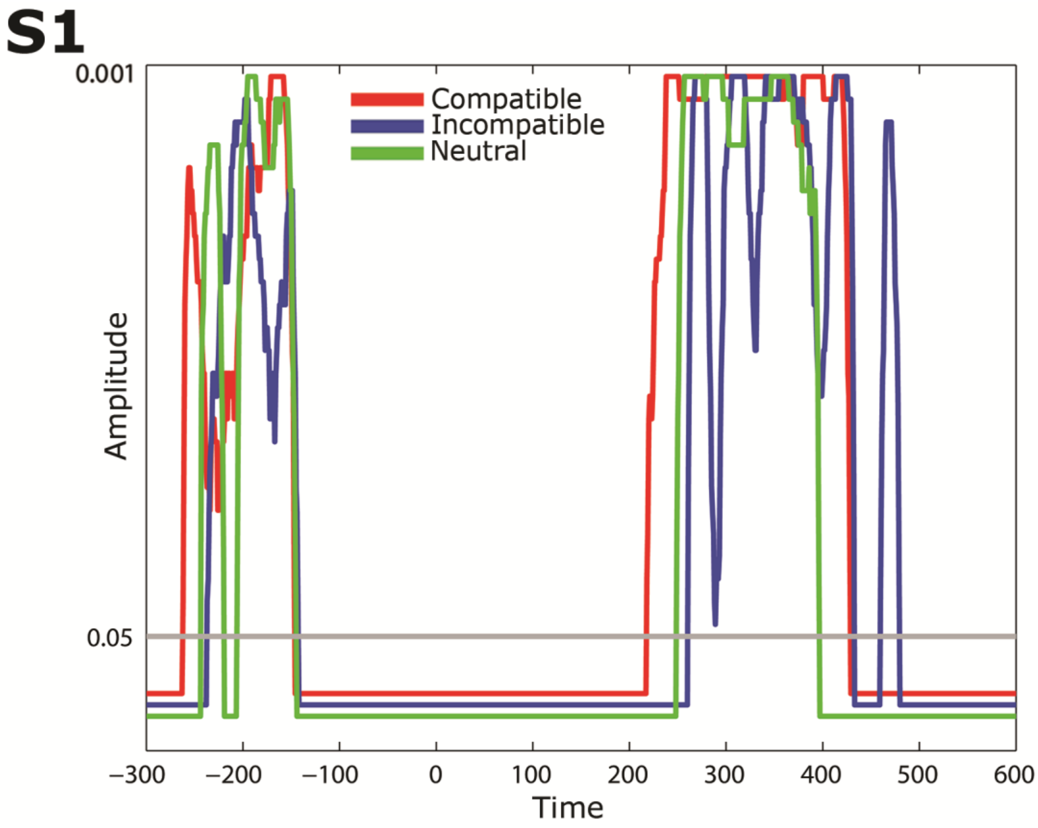

Figure S1. *p*-value for comparisons between EPD and controls in each condition. Significant differences were observed in the comparison for each condition among groups throughout the ERP time window (in the time windows preceding MP, -300 to 150 ms, and in the re-afferent potential time window, 220 to 480 ms). In each category comparison, the control group presented increased amplitudes compared with EPD patients (see Figure 1A).

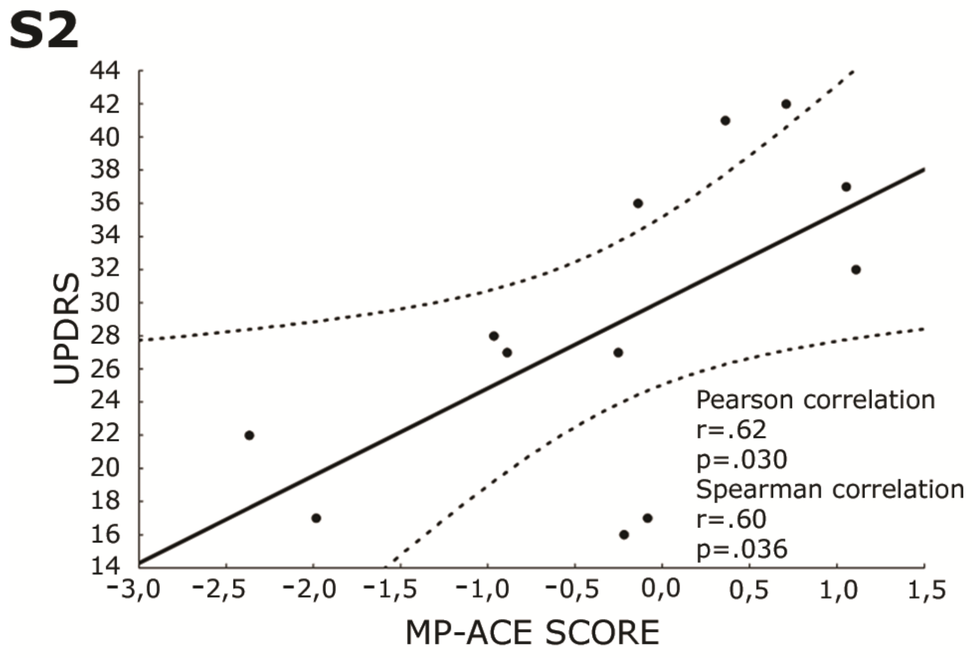

Figure S2: Correlation between UPDRS and MP-ACE score in EPD. The motor section of the UPDRS was associated with cortical measures of ACE in EPD (Pearson's r = .62, p = .030; Spearman's r = .60, p = .036). Note that two outlier values (> 3 SDs) were excluded from this analysis.

Table 1S: Mean and SD of each condition in both EPD patients and controls

Condition / Group	Compatible (Mean±SD)	Incompatible (Mean±SD)	Neutral (Mean±SD)
**EPD**	1522ms±701.50	1495ms±689.79	1462ms±804.98
**Controls**	805ms±255.52	1013ms±452.81	975ms±509.97

3. References

1 Hughes, A. J., Daniel, S. E., Kilford, L. & Lees, A. J. Accuracy of clinical diagnosis of idiopathic Parkinson's disease: a clinico-pathological study of 100 cases. *J Neurol Neurosurg Psychiatry*. **55**, 181-184 (1992).

2 Fahn, S. & Elton, R. L. in *Unified Parkinson´s disease rating scale.* (ed C. D. Marsden & M. Goldstein Fahn) 153-163 (Macmillan, 1987).

3 Hoehn, M. M. & Yahr, M. D. Parkinsonism: onset, progression and mortality. *Neurology*. **17**, 427-442 (1967).
4 Mattis, P. J., Tang, C. C., Ma, Y., Dhawan, V. & Eidelberg, D. Network correlates of the cognitive response to levodopa in Parkinson disease. *Neurology*. **77**, 858-865 (2011).
5 Lewis, S. J. *et al.* Heterogeneity of Parkinson's disease in the early clinical stages using a data driven approach. *J Neurol Neurosurg Psychiatry*. **76**, 343-348 (2005).
6 Williams-Gray, C. H., Hampshire, A., Barker, R. A. & Owen, A. M. Attentional control in Parkinson's disease is dependent on COMT val 158 met genotype. *Brain*. **131**, 397-408 (2008).
7 Wechsler, D. (Psychological Corporation, San Antonio, TX, 1999).
8 Bak, T. H. & Hodges, J. R. Kissing and dancing – A test to distinguish the lexical and conceptual contributions to noun/verb and action/object dissociation. Preliminary results in patients with frontotemporal dementia. *Journal of Neurolinguistics*. **16**, 169-181 (2003).
9 Badets, A. & Pesenti, M. Creating number semantics through finger movement perception. *Cognition*. **115**, 46-53 (2010).
10 Glenberg, A. M., Sato, M. & Cattaneo, L. Use-induced motor plasticity affects the processing of abstract and concrete language. *Curr Biol*. **18**, R290-291 (2008).
11 Linderman, W., Yanagida, Y., Norma, H. & Hosaka, K. Wereable vibrotactile systems for virtual contact and information display. *Virtual Reality*. **9**, 203-213 (2006).
12 Van Elk, M., Van Schie, H. T. & Bekkering, H. Semantics in action: An electrophysiological study on the use of semantic knowledge for action. *Journal of Physiology – Paris*. **102**, 95-100 (2008).
13 Aravena, P. *et al.* Applauding with closed hands: neural signature of action-sentence compatibility effects. *PLoS One*. **5**, e11751 (2010).
14 Manly, B. *Randomization, Bootstrap and Montecarlo Methods in Biology*. (1997).
15 Bandt, C. & Pompe, B. Permutation entropy: a natural complexity measure for time series. *Phys Rev Lett*. **88**, 174102 (2002).
16 King, J. R. *et al.* Information sharing in the brain indexes consciousness in noncommunicative patients. *Curr Biol*. **23**, 1914-1919 (2013).
17 Bertella, L. *et al.* Noun verb dissociation in Parkinson's disease. *Brain Cogn*. **48**, 277-280 (2002).
18 Cotelli, M. *et al.* Action and object naming in Parkinson's disease without dementia. *Eur J Neurol*. **14**, 632-637 (2007).
19 Ibanez, A. *et al.* Motor-language coupling: direct evidence from early Parkinson's disease and intracranial cortical recordings. *Cortex*. **49**, 968-984 (2013).
